# A Novel Gene Signature to Predict Survival Time and Incident Ventricular Arrhythmias in Patients with Dilated Cardiomyopathy

**DOI:** 10.1155/2020/8847635

**Published:** 2020-09-15

**Authors:** Chenliang Ge, Yan He

**Affiliations:** ^1^The First Affiliated Hospital of Guangxi Medical University, Nanning 530021, China; ^2^The First Affiliated Hospital of University of South China, Hengyang 421001 ., China

## Abstract

The mortality in nonischaemic dilated cardiomyopathy (NIDCM) patients is still at a high level; sudden death in NIDCM can be caused by ventricular tachycardia. It is necessary to explore the pathogenesis of ventricular arrhythmias (VA) in NIDCM. Differentially expressed genes (DEGs) were identified by comparing the gene expression of NIDCM patients with or without VA in the gene expression profile of GSE135055. A total of 228 DEGs were obtained, and 3 genes were screened out to be significantly related to the survival time of NIDCM patients. We established a prediction model on two-gene (*TOMM22*, *PPP2R5A*) signature for the survival time of NIDCM patients. The area under the curve (AUC) was 0.75 calculated by the ROC curve analysis. These risk genes are probably new targets for exploring the pathogenesis of NIDCM with VA; the prediction model for survival time and incident ventricular arrhythmias is useful in clinical decision making for individual treatment.

## 1. Introduction

Nonischaemic dilated cardiomyopathy (NIDCM) is one of the most common inherited cardiomyopathy and is considered to be one of the main causes of heart failure and sudden cardiac death. Heart transplantation is usually needed [1, 2]. One-third of patients with NIDCM may represent arrhythmogenic phenotypes and have an increased risk of arrhythmias during follow-up; ventricular arrhythmia (VA) is a major cause of clinical deterioration and demise in patients with NIDCM [3–6]. In order to prevent these situations, it is recommended that prophylactic intervention with implantable cardioverter-defibrillators (ICD) in patients with heart failure and left ventricular ejection fraction < 35% in current international guidelines [7, 8]. Although NIDCM is characterized by genetic and clinical heterogeneity, few studies have explored the pathogenesis of VA in patients with NIDCM. In order to reveal the inherent molecular mechanism, NIDCM patients were divided into sinus rhythm (SR) group and VA group and differentially expressed genes (DEGs) were identified via bioinformatic methods.

## 2. Materials and Methods

### 2.1. Data Source

We download the microarray expression dataset (GSE135055) from the Gene Expression Omnibus (GEO) database [9]. The multilevel transcriptional data of GSE135055 were generated from the heart tissues of 21 heart failure (HF) patients and 9 healthy donors. Among these HF patients, there are 18 NIDCM patients, during which 6 patients suffered VA, including ventricular premature beats, ventricular tachycardia, and ventricular fibrillation [10]. The characteristics of these patients are listed in Table 1 (supplemental file Table S1). We divided the NIDCM patients into two groups, with 12 patients in the sinus rhythm (SR) group and 6 patients in the VA group. We used the “limma” package [11] to quantile the datasets and screened out DEGs between the SR group and VA group with *p* < 0.05 and ∣log2FC | >1 were considered statistically significant. A volcano map and heat map were used to show the DEGs.

### 2.2. Construction of Survival Time Prediction Model

17 NIDCM patients (6 patients suffered VA included) with complete survival time (time period of each patient from symptoms to heart transplantation) information were selected for the study. By using univariate Cox regression analysis, we screened out DEG significantly related to survival time (*p* < 0.05) [12]. Then, a gene signature and a prediction model for survival time were constructed via multivariate cox analysis, survival analysis, and random survival forest algorithm.

### 2.3. Evaluation of Survival Time Prediction Model

Based on the regression coefficient and the expression value of each selected gene obtained by the multivariate Cox regression model, the risk score of each patient was calculated, then we separated 17 patients into high-risk and low-risk groups using the median risk score as the cutoff. A high-risk score indicates poor survival time for the NIDCM patients. The accuracy of the prediction model was evaluated by time-dependent ROC analysis.

### 2.4. Enrichment Analysis by Metascape

The pathway enrichment of DEGs was analyzed using Metascape. With the species limited to “Homo sapiens,” functional enrichment analysis was performed based on Gene Ontology, Kyoto Encyclopedia of Genes and Genomes, Reactive pathways, and Canonical Pathways [13]. All genes in the genome have been used as the enrichment background. Terms with a *p* value < 0.01, a minimum count of 3, and an enrichment factor > 1.5 are collected and grouped into clusters based on their membership similarities.

## 3. Results

### 3.1. Identification of Differentially Expressed Genes (DEGs)

**Figure 1:**
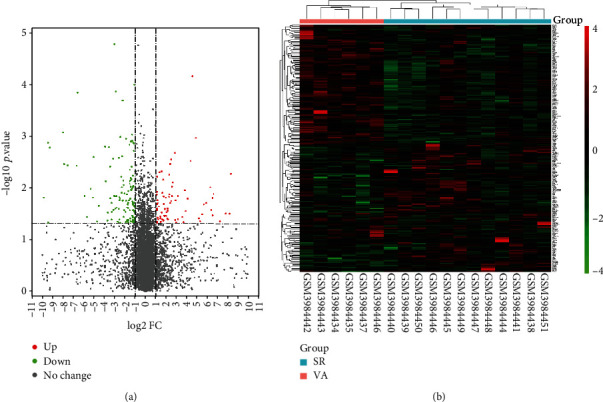
DEGs between SR and VA. (a) Volcano map of DEGs between SR and VA. Red represents upregulated differential genes, black represents no significant difference genes, and green represents downregulated differential genes. (b) Heat map of all DEGs between SR and VA. Each column represents a tissue sample, and each row represents a DEG. The gradual color change from green to red indicates the changing process from downregulation to upregulation.

A total of 228 DEGs were obtained via the microarray data analysis by Limma, including 86 upregulated and 142 downregulated DEG (supplemental file Table S2). A DEG expression volcano map and heat map were shown in Figure 1.

### 3.2. Identification of Survival-Related DEGs and Construction of Survival Time Prediction Model

**Figure 2:**
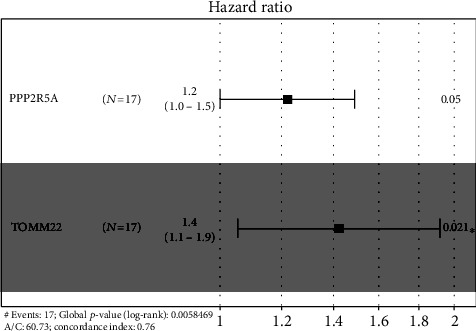
Forrest plot of the multivariate Cox regression analysis in NIDCM.

3 DEGs were screened out to be significantly related to the survival time of NIDCM patients via univariate Cox regression analysis including *TOMM22*, *TMSB4XP8*, and *PPP2R5A* (*p* < 0.05) (Table 2). The 3 genes were used to establish a model for predicting survival time of NIDCM patients. Multivariate Cox proportional hazard regression analysis was performed to confirm the optimal model and a forest map was shown in Figure 2. *PPP2R5A* and *TOMM22* were identified as risk genes in the survival time model. We confirmed *PPP2R5A* and *TOMM22* as high-risk genes in the survival time model which are negatively correlated with patient prognosis (Table 3).

### 3.3. Testing the Survival Time Prediction Model

**Figure 3:**
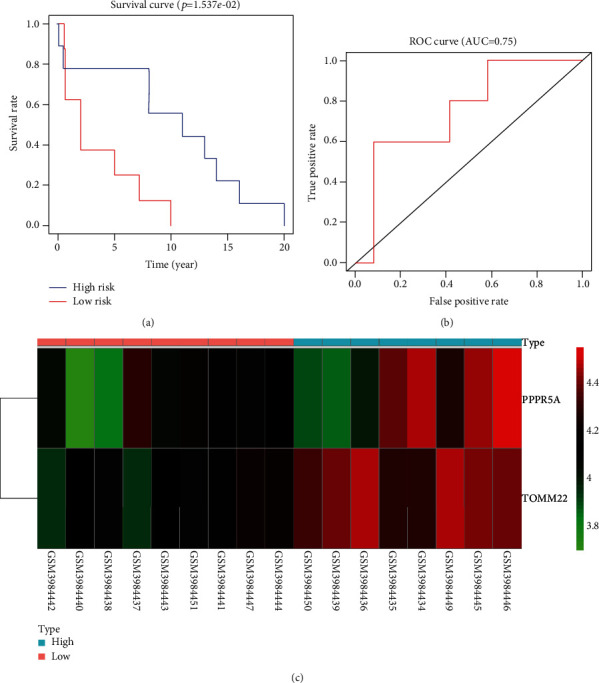
Validation of the survival time prognostic risk models in NIDCM patients. (a) Survival analysis of symptoms to heart transplantation in the high-risk (red line) and low-risk (blue line) NIDCM patients. (b) Time-dependent ROC curve analyses show AUC values for time from symptoms to heart transplantation in NIDCM patients. (c) Expression levels of risk genes in the high-risk and low-risk patients.

The prognostic risk scores for the 17 NIDCM patients were calculated using the gene expression values and the regression coefficients of the risk genes according to the prediction model: risk score for survival time = (0.198883 × expression value of *PPP*2*R*5*A*) + (0.350995 × expression value of *TOMM*22). Patients were subdivided into high and low-risk groups for survival time based on the median risk scores. Survival analysis revealed that high-risk scores were significantly related to poor survival time (symptoms to heart transplantation). The 2-year and 5-year survival rates for the high-risk patients were 37.5% and 25%, whereas the 8-year and 11-year survival rates for the low-risk patients were 55.6% and 44.4%. We then measured the predictive performance of the prognostic risk models using the time-dependent receiver operating characteristic (ROC) curves. The area under the curve (AUC) was 0.75, which indicated superior predictive accuracy in NIDCM patients for survival time (Figure 3).

### 3.4. Function Enrichment Analysis by Metascape

**Figure 4:**
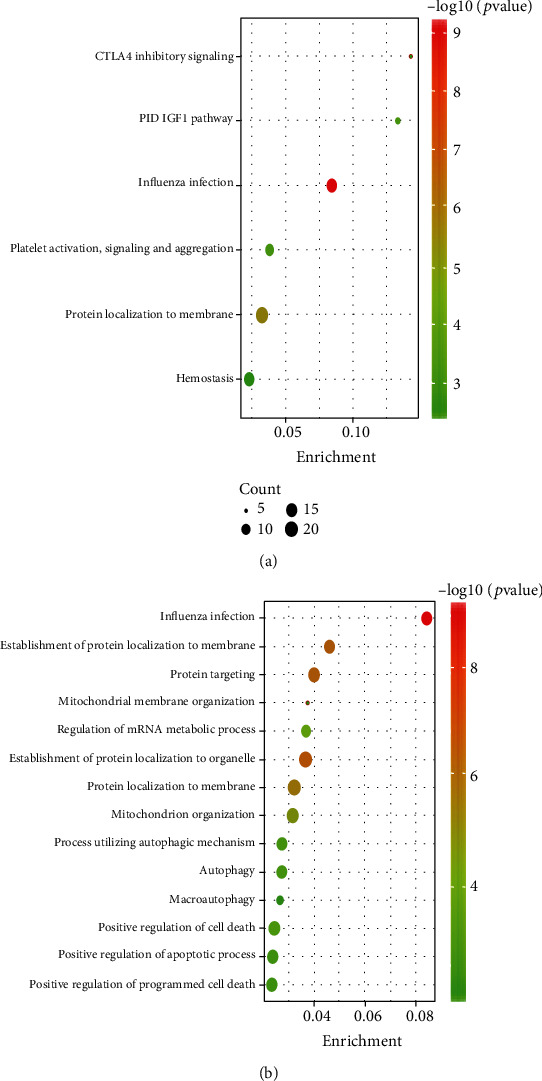
Enrichment analysis by Metascape. (a) Enriched terms of PPP2R5A. (b) Enriched terms of TOMM22.

The Metascape function enrichment results for DEGs including 204 Gene Ontology terms, 53 Reactome pathways, and 6 Kyoto Encyclopedia of Genes and Genomes pathways. The risk gene *PPP2R5A* is mainly involved in Influenza Infection, protein localization to membrane, PID IGF1 PATHWAY, CTLA4 inhibitory signaling, Platelet activation, signaling and aggregation, and Hemostasis. *TOMM22* is mainly enriched in Influenza Infection, establishment of protein localization to organelle, establishment of protein localization to membrane, protein targeting, protein localization to membrane, mitochondrion organization, autophagy, process utilizing autophagic mechanism, macroautophagy, regulation of mRNA metabolic process, and mitochondrial membrane organization (Figure 4).

## 4. Discussion

Due to high incidence, poor therapeutic effect, and prognosis, NIDCM is a major health problem that threatens human health, it is necessary to explore its pathogenesis and establish an accurate prediction prognosis model [14–16]. NIDCM is characterized as a genetically determined disease; the genetic basis of NIDCM highlights the importance of screening biomarkers for diagnosis and prognosis [17–19]. Although the survival time of patients with NIDCM is quite different [20, 21], there are few methods to predict the survival time after being diagnosed as NIDCM. We first established a novel two-gene signature to predict survival in patients with NIDCM. To our knowledge, the two-gene signature related prognostic model for NIDCM has not been reported previously. The risk score was based on mRNA expression but not somatic mutations or methylation status of only two prognostic genes, which could be more routine and cost-effective in practice.

VA is common in patients with NIDCM. Ventricular fibrillation and cardiac arrest are important reasons for death in patients with NIDCM [22, 23], while there have been few studies focusing solely on VA in NIDCM. Although sudden cardiac death rates have decreased, implantation of ICD for primary prevention in NIDCM patients does not provide overall survival benefits [24]. Establishing a prediction model for incident VA in patients with NIDCM helps make personalized treatment to improve the survival, which can be used as a reference index of whether patients need to be implanted with ICD or not. In this study, we established a novel two-gene signature (including *TOMM22* and *PPP2R5A*) to predict the occurrence of VA in patients. Consistent with our research, some studies support *PPP2R5A* as a novel target for the treatment of arrhythmia. *PPP2R*5A, protein phosphatase 2 regulatory subunit B'alpha, encodes an alpha isoform of the regulatory subunit B56 subfamily. Downregulated B56*α* myocytes are insensitive to isoproterenol-induced induction of arrhythmogenic Na^+^ channel late component. Voltage-gated Na^+^ channel 1.5 is critical for normal cardiac excitability, PP2A-B56*α* complex proved to interact with the primary cardiac voltage-gated Na^+^ channel 1.5 [25]. *TOMM22*, Translocase of outer mitochondrial membrane 22, is responsible for the recognition and translocation of synthesized mitochondrial precursor proteins; phosphorylation of *TOMM22* is a critical switch for mitophagy [26, 27]. Combined with the functional enrichment analysis results above, *TOMM22* may regulate mitochondrial autophagy in NIDCM, which needs to be confirmed by further research.

Tachycardia-induced cardiomyopathy (TIC) is characterized by diverse tachyarrhythmias, including supraventricular arrhythmias (such as atrial fibrillation) and ventricular arrhythmias [28, 29]. *PPP2R5A* and *TOMM22* show significantly different expression levels between the VA group and SR group in NIDCM patients, which suggests they may be related to the pathogenesis of TIC. More study needs to be done with *PPP2R5A* and *TOMM22* to determine its exact role in the pathogenesis of TIC.

However, there are some limitations in our research. We studied a small number of patients, although the ROC value (0.75) indicated superior predictive accuracy of the prediction model for survival time in NIDCM patients. In future research, we will carry out a large sample, randomized, and follow-up studies. Besides, the time period of each patient from symptoms to heart transplantation still can represent the prognosis of patients with NIDCM to some extent, overall survival data should be more accurate, and we will carry out patients' long-term follow-up to collect more detail clinical information.

## 5. Conclusions

Our study identified 3 new genes that were significantly related to the survival time of NIDCM and established a novel two-gene signature to predict survival time and incident VA of NIDCM. Although the sample size of our study is relatively small, these risk genes are probably new targets for exploring the pathogenesis of NIDCM with VA, and the prediction model is useful in clinical decision making for individual treatment.

## Figures and Tables

**Table 1:** Baseline parameters in NIDCM patients with and without ventricular arrhythmia.

Clinical features	SR (12)	VA (6)	*p* value
Age (y)	30.33 (16.87)	39.33 (16.17)	0.296
Male sex (*n* (%))	7 (58.3)	3 (50.0)	0.563
LA (mm)	41.08 (13.53)	35.33 (17.78)	0.454
LVEDD (mm)	68.00 (11.72)	58.33 (28.74)	0.318
LVEF (%)	27.00 (11.50)	24.00 (13.74)	0.631
AF (%)	2 (16.7)	2 (33.3)	0.407
NYHA functional class			
II	2 (16.7)	0 (0.0)	0.806
III	4 (33.3)	2 (33.3)	
IV	6 (50.0)	4 (66.7)	
Diabetes mellitus (%)	1 (8.3)	1 (16.7)	0.569
Smoking history (%)	2 (16.7)	1 (16.7)	0.755
Drug therapy			
Amiodarone (%)	1 (8.3)	2 (33.3)	0.245
*β*-Blockers (%)	8 (66.7)	4 (66.7)	0.691
Digoxin (%)	10 (83.3)	4 (66.7)	0.407
ACEI/ARB (%)	10 (83.3)	4 (66.7)	0.407
CCB (%)	2 (16.7)	1 (16.7)	0.755
Diuretic (%)	11 (91.7)	5 (83.3)	0.569

Values are mean ± SD, *n* (%); LA: left atrium; LVEDD: left ventricular diastolic diameter; LVEF: left ventricular ejection fraction; AF: Atrial fibrillation; NYHA: New York Heart Association; ACEI: angiotensin-converting enzyme inhibitors; ARB: angiotensin receptor blocker; CCB: calcium channel blocker.

**Table 2:** DEGs significantly related to survival time of NIDCM patients by univariate Cox regression analysis.

Gene symbol	HR	HR.95L	HR.95H	*p* value
TOMM22	1.42163	1.063455	1.900439	0.017531
TMSB4XP8	1.119692	1.019037	1.230289	0.018656
PPP2R5A	1.231818	1.009141	1.503629	0.040422

**Table 3:** Characteristics of risk DEGs in the prognostic risk models.

Gene symbol	Coef	HR	HR.95L	HR.95H	*p* value
PPP2R5A	0.198883	1.220039	0.999961	1.488553	0.050045
TOMM22	0.350995	1.42048	1.053479	1.915333	0.021358

## Data Availability

The dataset with mRNA expression profiling was taken from GEO: GSE135055. The associated corresponding clinical information of patients in this study was downloaded from BMC Medicine (doi:10.1186/s12916-019-1469-4).

## Abbreviations

NIDCM: Nonischaemic dilated cardiomyopathy
VA: Ventricular arrhythmias
GEO: Gene Expression Omnibus
DEGs: Differentially expressed genes
SR: Sinus rhythm
ROCs: Receiver operating characteristics
AUC: Area under the curve
HF: Heart failure.

## Supplementary Materials

Supplementary 1 Supplementary Table 1: summary of the basic information of DCM patients included in the study.

Supplementary 2 Supplementary Table 2: differentially expressed genes.
