# Supplementary material for: A Novel Gene Signature to Predict Survival Time and Incident Ventricular Arrhythmias in Patients with Dilated Cardiomyopathy
Source: Dis Markers. 2020 Sep 15;2020:8847635. doi: 10.1155/2020/8847635 (PMC7512094; doi:10.1155/2020/8847635)
Supplement: Supplementary 2 — Supplementary Table 2: differentially expressed genes. [file 8847635.f2.docx]

**Supplementary Table 2. Differentially expressed genes**

| **EntrezID** | **Gene Symbol** | **Description** | **logFC** | **t** | **P.Value** |
| --- | --- | --- | --- | --- | --- |
| 8557 | TCAP | titin-cap | 746.9467 | 2.838264 | 0.01151046 |
| 1937 | EEF1G | eukaryotic translation elongation factor 1 gamma | 208.9776 | 2.99528 | 0.008267702 |
| 1915 | EEF1A1 | eukaryotic translation elongation factor 1 alpha 1 | 119.283 | 2.501384 | 0.023107323 |
| 6191 | RPS4X | ribosomal protein S4 X-linked | 65.22853 | 3.420594 | 0.003334317 |
| 158078 | EEF1A1P5 | eukaryotic translation elongation factor 1 alpha 1 pseudogene 5 | 61.98023 | 2.480706 | 0.024099786 |
| 6136 | RPL12 | ribosomal protein L12 | 58.20361 | 3.107837 | 0.00651048 |
| 6194 | RPS6 | ribosomal protein S6 | 58.17838 | 3.033301 | 0.007627711 |
| 3043 | HBB | hemoglobin subunit beta | 40.61997 | 2.117731 | 0.049538984 |
| 2197 | FAU | FAU ubiquitin like and ribosomal protein S30 fusion | 34.45687 | 3.156006 | 0.005875511 |
| 6202 | RPS8 | ribosomal protein S8 | 33.76599 | 2.180899 | 0.043816886 |
| 6227 | RPS21 | ribosomal protein S21 | 33.5929 | 2.315516 | 0.033599974 |
| 6124 | RPL4 | ribosomal protein L4 | 31.93974 | 2.638001 | 0.017462216 |
| 427 | ASAH1 | N-acylsphingosine amidohydrolase 1 | 24.15448 | 2.46588 | 0.024836057 |
| 3040 | HBA2 | hemoglobin subunit alpha 2 | 18.02784 | 2.427044 | 0.026866651 |
| 6138 | RPL15 | ribosomal protein L15 | 10.74789 | 3.21171 | 0.005216813 |
| 6128 | RPL6 | ribosomal protein L6 | 8.316588 | 3.197772 | 0.005374481 |
| 6141 | RPL18 | ribosomal protein L18 | 8.184823 | 2.344418 | 0.031717651 |
| 54148 | MRPL39 | mitochondrial ribosomal protein L39 | 7.833244 | 2.345445 | 0.031652578 |
| 5705 | PSMC5 | proteasome 26S subunit, ATPase 5 | 7.27548 | 2.174405 | 0.044375793 |
| 22822 | PHLDA1 | pleckstrin homology like domain family A member 1 | 6.595557 | 2.775285 | 0.013131691 |
| 5478 | PPIA | peptidylprolyl isomerase A | 6.465818 | 2.69683 | 0.015460774 |
| 6695 | SPOCK1 | SPARC (osteonectin), cwcv and kazal like domains proteoglycan 1 | 6.443168 | 2.297287 | 0.034840214 |
| 2079 | ERH | ERH mRNA splicing and mitosis factor | 6.246142 | 2.925124 | 0.009588766 |
| 7135 | TNNI1 | troponin I1, slow skeletal type | 5.844585 | 2.584906 | 0.019479252 |
| 116362 | RBP7 | retinol binding protein 7 | 5.608678 | 2.562893 | 0.020379068 |
| 5093 | PCBP1 | poly(rC) binding protein 1 | 5.438885 | 2.314624 | 0.033659692 |
| 7117 | TMSB4XP8 | TMSB4X pseudogene 8 | 5.260471 | 2.165796 | 0.04512684 |
| 51142 | CHCHD2 | coiled-coil-helix-coiled-coil-helix domain containing 2 | 5.133616 | 2.227342 | 0.04000515 |
| 23633 | KPNA6 | karyopherin subunit alpha 6 | 4.849471 | 3.943546 | 0.001082589 |
| 56271 | BEX4 | brain expressed X-linked 4 | 4.525967 | 5.24338 | 7.03E-05 |
| 80755 | AARSD1 | alanyl-tRNA synthetase domain containing 1 | 4.048953 | 2.681623 | 0.015955969 |
| 3398 | ID2 | inhibitor of DNA binding 2 | 3.874516 | 2.389796 | 0.028960197 |
| 27238 | GPKOW | G-patch domain and KOW motifs | 3.763498 | 2.857412 | 0.011057124 |
| 5411 | PNN | pinin, desmosome associated protein | 3.657523 | 2.141807 | 0.047281723 |
| 51504 | TRMT112 | tRNA methyltransferase subunit 11-2 | 3.557576 | 2.259384 | 0.037556704 |
| 80142 | PTGES2 | prostaglandin E synthase 2 | 3.11696 | 2.209978 | 0.041392861 |
| 6150 | MRPL23 | mitochondrial ribosomal protein L23 | 2.936819 | 2.126083 | 0.048745022 |
| 9975 | NR1D2 | nuclear receptor subfamily 1 group D member 2 | 2.887785 | 2.643121 | 0.017278602 |
| 8106 | PABPN1 | poly(A) binding protein nuclear 1 | 2.860502 | 2.195247 | 0.042604911 |
| 5436 | POLR2G | RNA polymerase II subunit G | 2.833324 | 3.635283 | 0.002101239 |
| 6324 | SCN1B | sodium voltage-gated channel beta subunit 1 | 2.611638 | 3.02701 | 0.007730164 |
| 83444 | INO80B | INO80 complex subunit B | 2.540349 | 2.936553 | 0.00936035 |
| 610 | HCN2 | hyperpolarization activated cyclic nucleotide gated potassium and sodium channel 2 | 2.523761 | 2.735918 | 0.01425468 |
| 2339 | FNTA | farnesyltransferase, CAAX box, alpha | 2.46955 | 3.505284 | 0.002779422 |
| 677767 | SCARNA7 | small Cajal body-specific RNA 7 | 2.465505 | 2.63159 | 0.017694742 |
| 390354 | RPL18AP3 | ribosomal protein L18a pseudogene 3 | 2.361402 | 2.584997 | 0.019475614 |
| 1611 | DAP | death associated protein | 2.356789 | 3.406239 | 0.003438714 |
| 105378097 | LOC105378097 | uncharacterized LOC105378097 | 2.297369 | 2.769527 | 0.013290453 |
| 64110 | MAGEF1 | MAGE family member F1 | 2.264521 | 3.185753 | 0.005514186 |
| 406922 | MIR133A1 | microRNA 133a-1 | 2.242586 | 2.722275 | 0.014664997 |
| 23760 | PITPNB | phosphatidylinositol transfer protein beta | 2.168851 | 2.204049 | 0.041876852 |
| 131034 | CPNE4 | copine 4 | 2.145672 | 2.401669 | 0.028276779 |
| 6920 | TCEA3 | transcription elongation factor A3 | 2.113422 | 2.449687 | 0.025664479 |
| 51673 | TPPP3 | tubulin polymerization promoting protein family member 3 | 2.077143 | 2.215722 | 0.040928983 |
| 56993 | TOMM22 | translocase of outer mitochondrial membrane 22 | 2.031493 | 2.366661 | 0.030336659 |
| 22820 | COPG1 | COPI coat complex subunit gamma 1 | 1.995243 | 2.28272 | 0.035861817 |
| 91461 | PKDCC | protein kinase domain containing, cytoplasmic | 1.961626 | 2.706113 | 0.015165744 |
| 493753 | COA5 | cytochrome c oxidase assembly factor 5 | 1.871554 | 2.439427 | 0.026202795 |
| 51602 | NOP58 | NOP58 ribonucleoprotein | 1.835254 | 2.436134 | 0.026377807 |
| 11338 | U2AF2 | U2 small nuclear RNA auxiliary factor 2 | 1.829224 | 2.432739 | 0.026559394 |
| 91807 | MYLK3 | myosin light chain kinase 3 | 1.811452 | 2.276817 | 0.036283705 |
| 9194 | SLC16A7 | solute carrier family 16 member 7 | 1.78617 | 2.29004 | 0.035345047 |
| 7534 | YWHAZ | tyrosine 3-monooxygenase/tryptophan 5-monooxygenase activation protein zeta | 1.752132 | 2.569033 | 0.020124168 |
| 4675 | NAP1L3 | nucleosome assembly protein 1 like 3 | 1.741213 | 2.728554 | 0.01447476 |
| 6624 | FSCN1 | fascin actin-bundling protein 1 | 1.684227 | 2.174177 | 0.044395511 |
| 26802 | SNORD47 | small nucleolar RNA, C/D box 47 | 1.680334 | 2.18469 | 0.043493645 |
| 100859930 | HEIH | hepatocellular carcinoma up-regulated EZH2-associated long non-coding RNA | 1.674416 | 3.079368 | 0.0069169 |
| 6721 | SREBF2 | sterol regulatory element binding transcription factor 2 | 1.620982 | 2.805589 | 0.012325823 |
| 7905 | REEP5 | receptor accessory protein 5 | 1.60387 | 2.205997 | 0.041717269 |
| 100134934 | TEN1 | TEN1 subunit of CST complex | 1.554463 | 2.362061 | 0.030617564 |
| 114932 | MRFAP1L1 | Morf4 family associated protein 1 like 1 | 1.54273 | 3.250439 | 0.004802248 |
| 23277 | CLUH | clustered mitochondria homolog | 1.467346 | 2.149431 | 0.046586899 |
| 79365 | BHLHE41 | basic helix-loop-helix family member e41 | 1.421659 | 2.268412 | 0.036892333 |
| 670 | BPHL | biphenyl hydrolase like | 1.3221 | 2.495903 | 0.023366567 |
| 27106 | ARRDC2 | arrestin domain containing 2 | 1.276351 | 2.502803 | 0.023040675 |
| 26249 | KLHL3 | kelch like family member 3 | 1.205153 | 2.208657 | 0.041500247 |
| 9644 | SH3PXD2A | SH3 and PX domains 2A | 1.180375 | 2.58047 | 0.019657504 |
| 402057 | RPS17P16 | ribosomal protein S17 pseudogene 16 | 1.179085 | 2.242481 | 0.03883042 |
| 84701 | COX4I2 | cytochrome c oxidase subunit 4I2 | 1.165522 | 2.653424 | 0.016914746 |
| 5111 | PCNA | proliferating cell nuclear antigen | 1.136341 | 3.148308 | 0.005972743 |
| 10999 | SLC27A4 | solute carrier family 27 member 4 | 1.117091 | 2.834458 | 0.011602684 |
| 114926 | SMIM19 | small integral membrane protein 19 | 1.108234 | 2.410503 | 0.027778136 |
| 23002 | DAAM1 | dishevelled associated activator of morphogenesis 1 | 1.091804 | 2.563688 | 0.020345887 |
| 54494 | C11orf71 | chromosome 11 open reading frame 71 | 1.089612 | 2.121244 | 0.049203547 |
| 56984 | PSMG2 | proteasome assembly chaperone 2 | 1.085873 | 2.443064 | 0.026010777 |
| 55739 | NAXD | NAD(P)HX dehydratase | 1.073831 | 2.763606 | 0.013455652 |
| 5781 | PTPN11 | protein tyrosine phosphatase non-receptor type 11 | -1.01696 | -2.34452 | 0.031710993 |
| 9948 | WDR1 | WD repeat domain 1 | -1.02946 | -2.40177 | 0.028270974 |
| 9547 | CXCL14 | C-X-C motif chemokine ligand 14 | -1.03427 | -2.67447 | 0.016194107 |
| 2734 | GLG1 | golgi glycoprotein 1 | -1.03492 | -2.48392 | 0.023942717 |
| 9457 | FHL5 | four and a half LIM domains 5 | -1.06521 | -3.04679 | 0.007412556 |
| 7357 | UGCG | UDP-glucose ceramide glucosyltransferase | -1.06845 | -2.51006 | 0.022702461 |
| 55625 | ZDHHC7 | zinc finger DHHC-type palmitoyltransferase 7 | -1.08166 | -2.23603 | 0.039327389 |
| 9100 | USP10 | ubiquitin specific peptidase 10 | -1.09423 | -2.58783 | 0.01936249 |
| 5110 | PCMT1 | protein-L-isoaspartate (D-aspartate) O-methyltransferase | -1.09844 | -2.51817 | 0.022330196 |
| 55624 | POMGNT1 | protein O-linked mannose N-acetylglucosaminyltransferase 1 (beta 1,2-) | -1.09857 | -2.13788 | 0.047643797 |
| 5867 | RAB4A | RAB4A, member RAS oncogene family | -1.1042 | -5.05902 | 0.0001026 |
| 5770 | PTPN1 | protein tyrosine phosphatase non-receptor type 1 | -1.11004 | -2.37466 | 0.029853915 |
| 79647 | AKIRIN1 | akirin 1 | -1.11344 | -2.52587 | 0.021982328 |
| 23233 | EXOC6B | exocyst complex component 6B | -1.13516 | -2.18981 | 0.043060712 |
| 79810 | PTCD2 | pentatricopeptide repeat domain 2 | -1.13784 | -3.83031 | 0.001380973 |
| 23787 | MTCH1 | mitochondrial carrier 1 | -1.13901 | -2.1671 | 0.04501202 |
| 25777 | SUN2 | Sad1 and UNC84 domain containing 2 | -1.15504 | -3.68089 | 0.001904768 |
| 283871 | PGP | phosphoglycolate phosphatase | -1.16305 | -2.14766 | 0.046747421 |
| 57140 | RNPEPL1 | arginyl aminopeptidase like 1 | -1.18874 | -2.94738 | 0.009148954 |
| 23042 | PDXDC1 | pyridoxal dependent decarboxylase domain containing 1 | -1.19198 | -3.84756 | 0.001330681 |
| 1154 | CISH | cytokine inducible SH2 containing protein | -1.2045 | -2.41173 | 0.02770976 |
| 9077 | DIRAS3 | DIRAS family GTPase 3 | -1.20556 | -2.55307 | 0.020793106 |
| 54776 | PPP1R12C | protein phosphatase 1 regulatory subunit 12C | -1.22281 | -2.93227 | 0.009445274 |
| 54507 | ADAMTSL4 | ADAMTS like 4 | -1.22295 | -3.16208 | 0.005799898 |
| 1855 | DVL1 | dishevelled segment polarity protein 1 | -1.2247 | -2.59173 | 0.019208085 |
| 966 | CD59 | CD59 molecule (CD59 blood group) | -1.25674 | -2.24454 | 0.038672869 |
| 55323 | LARP6 | La ribonucleoprotein 6, translational regulator | -1.26441 | -2.18593 | 0.043388153 |
| 25822 | DNAJB5 | DnaJ heat shock protein family (Hsp40) member B5 | -1.27046 | -2.57767 | 0.01977092 |
| 55905 | RNF114 | ring finger protein 114 | -1.28591 | -2.62305 | 0.018008976 |
| 6611 | SMS | spermine synthase | -1.29166 | -2.28972 | 0.035367249 |
| 29979 | UBQLN1 | ubiquilin 1 | -1.29474 | -2.62712 | 0.017858696 |
| 3991 | LIPE | lipase E, hormone sensitive type | -1.29958 | -3.88576 | 0.001225739 |
| 80851 | SH3BP5L | SH3 binding domain protein 5 like | -1.31236 | -4.01538 | 0.000927855 |
| 9261 | MAPKAPK2 | MAPK activated protein kinase 2 | -1.31601 | -2.90202 | 0.010067206 |
| 1407 | CRY1 | cryptochrome circadian regulator 1 | -1.33575 | -3.35288 | 0.003855938 |
| 23381 | SMG5 | SMG5 nonsense mediated mRNA decay factor | -1.34713 | -2.8683 | 0.010807041 |
| 54583 | EGLN1 | egl-9 family hypoxia inducible factor 1 | -1.35617 | -3.08731 | 0.006801098 |
| 113829 | SLC35A4 | solute carrier family 35 member A4 | -1.37535 | -2.86795 | 0.010815159 |
| 6272 | SORT1 | sortilin 1 | -1.39923 | -3.87331 | 0.001258984 |
| 57552 | NCEH1 | neutral cholesterol ester hydrolase 1 | -1.42224 | -2.16146 | 0.04550923 |
| 145482 | PTGR2 | prostaglandin reductase 2 | -1.44737 | -2.93681 | 0.00935529 |
| 94005 | PIGS | phosphatidylinositol glycan anchor biosynthesis class S | -1.44938 | -3.11905 | 0.006356964 |
| 51696 | HECA | hdc homolog, cell cycle regulator | -1.46895 | -2.68085 | 0.015981462 |
| 207 | AKT1 | AKT serine/threonine kinase 1 | -1.47033 | -3.06374 | 0.007150464 |
| 2137 | EXTL3 | exostosin like glycosyltransferase 3 | -1.47657 | -2.22558 | 0.040143604 |
| 8724 | SNX3 | sorting nexin 3 | -1.52125 | -2.41029 | 0.027790013 |
| 64129 | TINAGL1 | tubulointerstitial nephritis antigen like 1 | -1.52325 | -2.99159 | 0.00833246 |
| 50999 | TMED5 | transmembrane p24 trafficking protein 5 | -1.5299 | -3.28858 | 0.004425728 |
| 64073 | C19orf33 | chromosome 19 open reading frame 33 | -1.53028 | -3.05126 | 0.007342537 |
| 55062 | WIPI1 | WD repeat domain, phosphoinositide interacting 1 | -1.54798 | -3.80391 | 0.001461694 |
| 79699 | ZYG11B | zyg-11 family member B, cell cycle regulator | -1.60012 | -2.64195 | 0.017320406 |
| 84248 | FYTTD1 | forty-two-three domain containing 1 | -1.65578 | -2.18941 | 0.043094102 |
| 10865 | ARID5A | AT-rich interaction domain 5A | -1.68773 | -2.84529 | 0.011342077 |
| 5887 | RAD23B | RAD23 homolog B, nucleotide excision repair protein | -1.69476 | -2.14358 | 0.047119406 |
| 653513 | LOC653513 | phosphodiesterase 4D interacting protein-like | -1.70527 | -2.59327 | 0.019147385 |
| 374 | AREG | amphiregulin | -1.70885 | -2.46052 | 0.025107648 |
| 7326 | UBE2G1 | ubiquitin conjugating enzyme E2 G1 | -1.7249 | -2.55639 | 0.020652476 |
| 10521 | DDX17 | DEAD-box helicase 17 | -1.76361 | -2.1917 | 0.042901304 |
| 388403 | YPEL2 | yippee like 2 | -1.77692 | -2.76972 | 0.013285028 |
| 10133 | OPTN | optineurin | -1.79141 | -2.16494 | 0.045202161 |
| 51582 | AZIN1 | antizyme inhibitor 1 | -1.80578 | -2.48059 | 0.024105671 |
| 90411 | MCFD2 | multiple coagulation factor deficiency 2, ER cargo receptor complex subunit | -1.88078 | -2.6364 | 0.017520178 |
| 5045 | FURIN | furin, paired basic amino acid cleaving enzyme | -1.88196 | -3.92773 | 0.001120012 |
| 55571 | CNOT11 | CCR4-NOT transcription complex subunit 11 | -1.90935 | -2.14474 | 0.047012887 |
| 84700 | MYO18B | myosin XVIIIB | -1.92936 | -2.3702 | 0.03012245 |
| 57538 | ALPK3 | alpha kinase 3 | -2.031 | -2.79507 | 0.012599898 |
| 2963 | GTF2F2 | general transcription factor IIF subunit 2 | -2.08209 | -2.40943 | 0.027838131 |
| 3840 | KPNA4 | karyopherin subunit alpha 4 | -2.11701 | -2.96987 | 0.008724326 |
| 5033 | P4HA1 | prolyl 4-hydroxylase subunit alpha 1 | -2.12747 | -2.26361 | 0.037244156 |
| 8733 | GPAA1 | glycosylphosphatidylinositol anchor attachment 1 | -2.15983 | -3.0641 | 0.007144935 |
| 84272 | YIPF4 | Yip1 domain family member 4 | -2.21494 | -2.64017 | 0.017384211 |
| 4783 | NFIL3 | nuclear factor, interleukin 3 regulated | -2.27609 | -3.56669 | 0.002435487 |
| 131566 | DCBLD2 | discoidin, CUB and LCCL domain containing 2 | -2.29083 | -2.92509 | 0.009589514 |
| 139322 | APOOL | apolipoprotein O like | -2.30655 | -2.80681 | 0.012294364 |
| 11057 | ABHD2 | abhydrolase domain containing 2, acylglycerol lipase | -2.33683 | -3.14362 | 0.006032733 |
| 7079 | TIMP4 | TIMP metallopeptidase inhibitor 4 | -2.41647 | -2.1209 | 0.049236206 |
| 51076 | CUTC | cutC copper transporter | -2.43324 | -3.96423 | 0.001035556 |
| 29984 | RHOD | ras homolog family member D | -2.50011 | -2.68089 | 0.015980112 |
| 5525 | PPP2R5A | protein phosphatase 2 regulatory subunit B'alpha | -2.53372 | -2.64754 | 0.017121798 |
| 3839 | KPNA3 | karyopherin subunit alpha 3 | -2.541 | -2.20306 | 0.041957772 |
| 8303 | SNN | stannin | -2.55167 | -2.7148 | 0.014894746 |
| 100127983 | C8orf88 | chromosome 8 open reading frame 88 | -2.57836 | -3.53721 | 0.002594973 |
| 51719 | CAB39 | calcium binding protein 39 | -2.58182 | -2.1267 | 0.048687185 |
| 210 | ALAD | aminolevulinate dehydratase | -2.5937 | -2.43669 | 0.02634831 |
| 9513 | FXR2 | FMR1 autosomal homolog 2 | -2.72329 | -2.15839 | 0.045782579 |
| 133 | ADM | adrenomedullin | -2.7808 | -2.47809 | 0.02422811 |
| 6196 | RPS6KA2 | ribosomal protein S6 kinase A2 | -2.83006 | -2.73329 | 0.014332751 |
| 3679 | ITGA7 | integrin subunit alpha 7 | -2.87093 | -4.92011 | 0.000136771 |
| 22800 | RRAS2 | RAS related 2 | -2.89961 | -2.45234 | 0.025526888 |
| 9805 | SCRN1 | secernin 1 | -3.00251 | -5.95948 | 1.68E-05 |
| 7483 | WNT9A | Wnt family member 9A | -3.00897 | -2.74131 | 0.014095557 |
| 1613 | DAPK3 | death associated protein kinase 3 | -3.09462 | -2.41973 | 0.027266211 |
| 9778 | KIAA0232 | KIAA0232 | -3.10283 | -2.42352 | 0.02705871 |
| 4200 | ME2 | malic enzyme 2 | -3.13409 | -3.5149 | 0.002722521 |
| 10150 | MBNL2 | muscleblind like splicing regulator 2 | -3.26622 | -2.37024 | 0.030119489 |
| 10231 | RCAN2 | regulator of calcineurin 2 | -3.27775 | -2.20273 | 0.041984924 |
| 11344 | TWF2 | twinfilin actin binding protein 2 | -3.28504 | -2.58837 | 0.019341097 |
| 10486 | CAP2 | cyclase associated actin cytoskeleton regulatory protein 2 | -3.41392 | -2.69157 | 0.015630236 |
| 6345 | SRL | sarcalumenin | -3.42217 | -3.35343 | 0.003851429 |
| 55664 | CDC37L1 | cell division cycle 37 like 1 | -3.54036 | -3.7547 | 0.001624947 |
| 26994 | RNF11 | ring finger protein 11 | -3.62271 | -2.35606 | 0.030987524 |
| 2632 | GBE1 | 1,4-alpha-glucan branching enzyme 1 | -3.67584 | -2.17571 | 0.044262774 |
| 7465 | WEE1 | WEE1 G2 checkpoint kinase | -3.84024 | -3.04859 | 0.007384307 |
| 1729 | DIAPH1 | diaphanous related formin 1 | -3.89795 | -3.77096 | 0.001569092 |
| 5236 | PGM1 | phosphoglucomutase 1 | -4.52914 | -2.52995 | 0.021799944 |
| 160760 | PPTC7 | protein phosphatase targeting COQ7 | -4.67995 | -2.1608 | 0.04556808 |
| 112399 | EGLN3 | egl-9 family hypoxia inducible factor 3 | -5.00863 | -3.55138 | 0.002517054 |
| 27122 | DKK3 | dickkopf WNT signaling pathway inhibitor 3 | -5.07303 | -2.70894 | 0.015076898 |
| 64094 | SMOC2 | SPARC related modular calcium binding 2 | -5.28635 | -2.88595 | 0.0104135 |
| 64397 | ZNF106 | zinc finger protein 106 | -5.6652 | -2.26102 | 0.037435306 |
| 2997 | GYS1 | glycogen synthase 1 | -5.87994 | -2.74334 | 0.014036153 |
| 55827 | DCAF6 | DDB1 and CUL4 associated factor 6 | -6.55572 | -4.89391 | 0.000144423 |
| 5738 | PTGFRN | prostaglandin F2 receptor inhibitor | -6.65774 | -3.3694 | 0.003721706 |
| 7414 | VCL | vinculin | -7.51161 | -3.37909 | 0.003645062 |
| 8637 | EIF4EBP3 | eukaryotic translation initiation factor 4E binding protein 3 | -7.85606 | -3.40183 | 0.003471406 |
| 51300 | TIMMDC1 | translocase of inner mitochondrial membrane domain containing 1 | -7.94609 | -4.06416 | 0.000835689 |
| 10272 | FSTL3 | follistatin like 3 | -9.23582 | -3.74538 | 0.001657884 |
| 4653 | MYOC | myocilin | -9.40072 | -2.13249 | 0.048143741 |
| 3856 | KRT8 | keratin 8 | -9.40508 | -3.84814 | 0.001329016 |
| 7532 | YWHAG | tyrosine 3-monooxygenase/tryptophan 5-monooxygenase activation protein gamma | -9.83724 | -2.7052 | 0.015194545 |
| 10063 | COX17 | cytochrome c oxidase copper chaperone COX17 | -10.566 | -2.18902 | 0.043126697 |
| 1264 | CNN1 | calponin 1 | -10.5734 | -3.22767 | 0.005041869 |
| 3074 | HEXB | hexosaminidase subunit beta | -11.8444 | -2.44439 | 0.0259412 |
| 2273 | FHL1 | four and a half LIM domains 1 | -12.0229 | -4.07936 | 0.000808898 |
| 23710 | GABARAPL1 | GABA type A receptor associated protein like 1 | -12.2836 | -2.18277 | 0.043656681 |
| 165904 | XIRP1 | xin actin binding repeat containing 1 | -12.4802 | -2.85433 | 0.011128917 |
| 4946 | OAZ1 | ornithine decarboxylase antizyme 1 | -13.9639 | -2.74129 | 0.014096162 |
| 9553 | MRPL33 | mitochondrial ribosomal protein L33 | -15.9397 | -2.66552 | 0.016497012 |
| 10324 | KLHL41 | kelch like family member 41 | -16.3227 | -2.48735 | 0.023776382 |
| 10449 | ACAA2 | acetyl-CoA acyltransferase 2 | -16.5567 | -2.64127 | 0.017344766 |
| 100131138 | LINC01405 | long intergenic non-protein coding RNA 1405 | -20.8829 | -4.11777 | 0.000745007 |
| 51330 | TNFRSF12A | TNF receptor superfamily member 12A | -22.134 | -2.51243 | 0.022593038 |
| 7388 | UQCRH | ubiquinol-cytochrome c reductase hinge protein | -25.588 | -2.94197 | 0.009253999 |
| 88 | ACTN2 | actinin alpha 2 | -33.1967 | -2.84064 | 0.011453126 |
| 26287 | ANKRD2 | ankyrin repeat domain 2 | -33.9213 | -3.99318 | 0.000973146 |
| 3312 | HSPA8 | heat shock protein family A (Hsp70) member 8 | -35.0692 | -2.19201 | 0.042875414 |
| 51778 | MYOZ2 | myozenin 2 | -45.1148 | -2.22377 | 0.040287183 |
| 4541 | ND6 | NADH dehydrogenase, subunit 6 (complex I) | -72.4134 | -2.60827 | 0.018565721 |
| 4023 | LPL | lipoprotein lipase | -90.7153 | -2.11525 | 0.049777544 |
| 347 | APOD | apolipoprotein D | -96.943 | -5.11859 | 9.08E-05 |
| 10627 | MYL12A | myosin light chain 12A | -138.014 | -2.3035 | 0.034412611 |
| 126393 | HSPB6 | heat shock protein family B (small) member 6 | -232.125 | -3.1536 | 0.005905769 |
